# OPTIMIZING VOLUNTARY WORK DESIGN FOR HEALTHY AGING: RETHINKING VOLUNTEER ENGAGEMENT

**DOI:** 10.1093/geroni/igae098.4247

**Published:** 2024-12-31

**Authors:** Shiyu Lu

**Affiliations:** The University of Hong Kong, Hong Kong, China (People’s Republic)

## Abstract

While existing research primarily focuses on the health benefits of volunteering in terms of quantity (e.g., participation or volunteer hours), the psychosocial aspects of role engagement in volunteering and its impact on healthy aging remain underexplored. Additionally, insights on establishing a supportive volunteering environment at the organizational level to facilitate healthy aging are lacking. This innovative study connected voluntary work design, role engagement in volunteering, and subjective well-being (SWB) among older adults. In an ongoing cross-sectional study involving 258 volunteers aged 60 and above in Hong Kong, the research revealed that the role engagement of volunteers (β = 0.08, p = 0.014) significantly correlated with SWB, while no significant association was observed between volunteer hours in the past 12 months and SWB (β = 0.001, p = 0.297), after adjusting for factors like age, gender, and cognitive function. Introducing elements of voluntary work design, such as task significance, organizational support, and autonomy, into the model unveiled a direct link between organizational support and improved SWB (β = 0.15, p = 0.003). Task significance (0.32, p =0.028) and organizational support (β = 0.04, p = 0.016) were further found to influence SWB through the role engagement of volunteering. The study underscores both theoretical and practical implications concerning the creation of a supportive volunteering environment conducive to healthy aging.

